# Evaluation of single-cell classifiers for single-cell RNA sequencing data sets

**DOI:** 10.1093/bib/bbz096

**Published:** 2019-10-23

**Authors:** Xinlei Zhao, Shuang Wu, Nan Fang, Xiao Sun, Jue Fan

**Affiliations:** 1 State Key Laboratory of Bioelectronics, Biomedical Engineering School, Southeast University, Nanjing 210096, China; 2 Singleron Biotechnologies, Nanjing 211800, China

**Keywords:** single-cell RNA-seq, classification, comparative analysis, benchmark

## Abstract

Single-cell RNA sequencing (scRNA-seq) has been rapidly developing and widely applied in biological and medical research. Identification of cell types in scRNA-seq data sets is an essential step before in-depth investigations of their functional and pathological roles. However, the conventional workflow based on clustering and marker genes is not scalable for an increasingly large number of scRNA-seq data sets due to complicated procedures and manual annotation. Therefore, a number of tools have been developed recently to predict cell types in new data sets using reference data sets. These methods have not been generally adapted due to a lack of tool benchmarking and user guidance. In this article, we performed a comprehensive and impartial evaluation of nine classification software tools specifically designed for scRNA-seq data sets. Results showed that Seurat based on random forest, SingleR based on correlation analysis and CaSTLe based on XGBoost performed better than others. A simple ensemble voting of all tools can improve the predictive accuracy. Under nonideal situations, such as small-sized and class-imbalanced reference data sets, tools based on cluster-level similarities have superior performance. However, even with the function of assigning ‘unassigned’ labels, it is still challenging to catch novel cell types by solely using any of the single-cell classifiers. This article provides a guideline for researchers to select and apply suitable classification tools in their analysis workflows and sheds some lights on potential direction of future improvement on classification tools.

## Introduction

Categorizing cell identity is an essential step to have a comprehensive knowledge of the composition of human organs and tissues, which is also the foundation to further explore the cell basis of human diseases. Conventionally, techniques such as immunohistochemistry [1], fluorescence-activated cell sorting (FACS) [2, 3] and morphological methods [4] are used to identify cell types. With the rapid development of single-cell separation and sequencing technologies [5–11], researchers can now easily obtain a large scale of gene expression profiles of individual cells, hence characterizing the cell types and functions of single cells in an unbiased manner [12].

A typical single-cell RNA sequencing (scRNA-seq) analysis workflow implements cell clustering and then cluster-based cell type identification using canonical cell type markers. However, there are several limitations to this strategy. First, clustering results, such as the number of clusters, largely depend on analysis tools and chosen parameters used for each tool. Researchers might have to test multiple clustering tools and multiple parameters for each data set to obtain a consensus result. Second, the results of clustering are sensitive to the number of cells in the data sets. Meanwhile, the time and memory consumption increase exponentially with the increasing number of cells. Third, it requires expert knowledge of canonical cell type markers to enable the identification of cell types. Finally, the manual annotation process is labor intensive. Sometimes, it even requires an iterative process between clustering parameter tuning and cell type assignment.

With more and more studies published and large-scale survey studies of mouse [10, 13] and human [14] becoming available, cell types in both normal and disease tissues [15, 16] are accumulated in great quantities. Gradually, scRNA-seq study designs have been shifting from discovering new cell types to becoming a high-resolution assay to profile subtle changes of cell type proportions and cell type-specific expression signatures, for example, different treatment responses between patient subgroups [17]. Therefore, it is now both feasible and inevitable to apply classification techniques for categorizing single cells into known cell types based on annotated public data sets. Reference data set-based cell type annotation does not require domain knowledge of the cell types, and time consumption linearly increases with the scale of test data sets with the possible parallelization.

A rapidly growing list of single-cell classification tools has been developed specifically for scRNA-seq data over the past 2 years [18]. These tools can be loosely divided into two categories. One assigns cell types based on its nearest neighbors using similarity measurements, such as scMCA [10], using Pearson correlation and scmap [19] adopting cosine similarity. This type of the tools usually has a preset model with tunable parameters, and the neighbors could either be on the cell level or on the cluster level. The other is developed with supervised learning algorithms, such as scPred [20] based on support vector machine (SVM) by default and Seurat [7, 21] based on random forest (RF). Both types of tools predict cell type labels of a new data set based on a reference data set. Single-cell label prediction is a relatively new approach, unlike other analysis components in the scRNA-seq workflow such as differential expression [22, 23], clustering [24, 25], trajectory inference [26] and imputation [27], and has not been evaluated systematically and comprehensively. Some published reports showed comparison analysis between different classification tools such as CellFishing compared with scmap [28], but the results are still limited. Without a benchmark study to compare all available tools, it is difficult for researchers to choose an appropriate tool and incorporate it into their workflow.

In this article, nine tools listed in the ‘classification’ category on scrna-tools.org [18] have been systematically compared. Internally generated mixed cell line data sets and a few public data sets with various complexities, all with well-annotated cell type labels, are used to test their performances. We first tested these tools on eight pairs of reference/test data sets with default parameters to evaluate their baseline performances. We then assessed the stability of the performances by altering the number of reference cells and by randomly sampling reference cells from the same data set. Next, we explored risk–benefit balance when these tools have the capability to identify novel cell types. Finally, we analyzed the performances of all tools when reference cell types have imbalanced cell numbers. We applied widely used evaluation metrics, such as accuracy, receiver operator characteristic (ROC) curves and area under ROC curves (AUC), to estimate the performances of the classification tools. We believe this work would provide guidance on choosing supervised cell type classification tools for scRNA-seq data sets under various user scenarios. We also presented suggestions on potential directions of future classification tool development.

## Methods

### Data

scRNA-seq data sets with well-annotated cell labels are required for a comprehensive and systematic evaluation of single-cell classification tools, since calculations of most evaluation metrics rely on a ground truth label set. Therefore, we only included scRNA-seq data sets with highly credible cell type labels. In this article, the following three sources of scRNA-seq data sets were used.

#### Mixed Cell Lines

Cell lines are rather homogenous populations. Clustering-based approaches could generate near-truth cell type labels of mixed cell line data when the number of clusters is known [25]. We generated two scRNA-seq data sets of mixed cell lines in house as described below. Three human cell lines, K562, HEK293T and A431, and one murine cell line, L929, were cultured separately in DMEM (Thermo Fisher Gibco), with 10% fetal bovine serum (Thermo Fisher Gibco) and 1% penicillin–streptomycin (Beyotime Biotechnology) in an incubator with 5% carbon dioxide at 37 °C. Single-cell suspensions with 1 × 10^5^ cells/ml in concentration in phosphate buffered saline (HyClone) were prepared. Two experiments were conducted. One is named as Mix3, where K562, 293T and L929 cell suspensions were mixed in 1:1:1 ratio. The other is named as Mix4, where cell suspensions of all four cell lines were mixed in 1:1:1:1 ratio. Mixed single-cell suspensions were then loaded onto microfluidic devices, and scRNA-seq libraries were constructed following Singleron GEXSCOPE™ protocol [29] using GEXSCOPE™ Single-Cell RNA Library Kit (Singleron Biotechnologies). Sequencing was performed on Illumina HiSeq X with 150 bp paired end reads to obtain a sequencing depth of approximately 6.5 K reads/cell.

Raw reads were processed to generate gene expression profiles using an internal pipeline. Briefly, after filtering read 1 without polyT tails, cell barcode and unique molecule identifier (UMI) were extracted. Adapters and polyA tails were trimmed before read 2 was mapped to GRCh38 and mm10 reference genome with ensemble version 92 gene annotation. Reads with the same cell barcode, UMI and gene were grouped together to calculate the number of UMIs per gene per cell. Cell number was then determined based on the ‘knee’ method. We used Seurat to perform clustering analysis for Mix3 and Mix4 data sets separately using FindCluster function with resolution equal to 0.1, to generate three and four clusters, respectively. The clusters are well separated as shown by the t-distributed stochastic neighbor embedding (t-SNE) [30] plots (Supplementary Figure S1A and C). Differential expression analysis with default parameters was conducted to find marker genes per cluster. Top 10 marker genes per cluster are shown in Supplementary Figure S1B and D. A431 cells were identified by gene KRT7. HEK293T cells were identified by SOX4 and K562 cells were identified by HBA1. The murine cell line L929 was identified by mouse gene names. The cell numbers of each cell line in two experiments are shown in Supplementary Table S1.

#### Peripheral blood mononuclear cell

Human peripheral blood mononuclear cell (PBMC) samples are easy to obtain and routinely studied in the fields such as immunology and infectious diseases. They are extremely heterogeneous populations containing a mixture of a hierarchical structure of cell types and subtypes. Here, we used PBMC scRNA-seq data sets generated by the 10× Genomics GemCode protocol [9]. The authors purified 10 PBMC subpopulations by antibody-based bead enrichment and further confirmed the cell identities by FACS sorting. The 10 populations were then individually processed to generate their single-cell gene expression profiles. In this article, UMI counts per cell of 10 presorted and filtered cell types of PBMC (detailed information in Supplementary Table S2) were downloaded. We later combined them into different data sets for different evaluation purposes.

#### Human pancreas data sets

Three public scRNA-seq data sets of human pancreas were used in this article to evaluate the classification tools (Supplementary Tables S3 and S4). They were generated using different experimental protocols in different labs and are from the same organ of different individuals. They have been widely used in the publications of many single-cell classification tools [19, 20, 28]. Hence, we used them to model the most realistic situation where a new data set is projected to an annotated data set from the same tissue. We downloaded Bioconductor SingleCellExperiment class objects of these data sets converted by Herberg’s lab with cell type annotations [19]. Cells with low quality or unknown cell labels were removed, such as ‘unclear’ in the Muraro data set and ‘alpha.contaminated’, ‘beta.contaminated’, ‘gamma.contaminated’ and ‘delta.contaminated’ in the Xin data set.

### Classification tools

In this study, we aimed to evaluate all tools in the classification category of the website scrna-tools.org [18] before 31 December 2018 (Table 1 and Supplementary Table S5). Some tools adopted widely used supervised learning algorithms, such as K-Nearest Neighbor (KNN), RF and SVM. Other tools are based on cluster-level similarity measurements, such as Pearson correlation in scMCA and Spearman correlation in SingleR [31], which calculate the similarity between query cells and one representative gene expression per cell type in reference data sets. All tools require both gene expression matrix and corresponding cell type annotations for reference data input and only the former for test data input.

**Table 1:** Classification tools selected for evaluation in this article and their information

Tools		(Pre)published date	Feature selection	Algorithm	Data format	‘Unassigned’ function
Scmap	scmapc2c	02/04/2018	Yes#	*k*-Means and approximate KNN, cosine distance	Normalized counts/log counts	Yes
	scmapc2clus			cluster-level median expression, cosine distance		
scMCA		02/22/2018	No	Cluster-level mean expression, Pearson correlation	Log counts	No
scPred		07/14/2018*	Yes	SVM	Normalized counts → cpm	Yes
SingleR		01/14/2019	Yes#	Cluster-level median expression, Spearman correlation	(Normalized) counts	No
Seurat		04/13/2015	Yes	RandomForest	(Normalized) counts	No
CaSTLe		10/10/2018	Yes	XGBoost	Log counts	No
scID		11/14/2018*	Yes#	A two-mixed Gaussian distribution	Counts → norm counts (with build-in function)	Yes
AltAnalyze		08/31/2016	Yes#	No description for algorithm	Norm counts	No
CellFishing		11/29/2018	Yes#	approximate k-NN, Locality- sensitive hashing and Hamming distance	Raw counts	No

*Note.* Dates labeled with ‘*’ mean the preprint date of the corresponding tool. The # label means corresponding tools have the option to perform the feature selection using a user-defined gene list. The published date of Seurat and AltAnalyze are the published dates for the packages but not for their classification functions. The version of all tools adopted in this article was up to date as of 31 December 2018.

The scmap [19] package contains two variations: scmapCluster and scmapCell. scmapCluster first constructs a virtual representation of each cell type in reference data set by extracting the median value of each feature (namely gene). It then calculates the similarity between each query cell and all cell type-specific virtual cells. The label of the query cell is assigned as the cell type of the virtual cell with the highest similarity. scmapCell directly calculates the similarity between the query cell and all of the reference cells. It then labels the query cell if the similarity exceeds a threshold and the *k* nearest neighbors are from the same cell type. scmapCluster and scmapCell are referred as scmapc2clus and scmapc2c, evaluated as separate tools in this article. The published version of scMCA [10] does not support user-provided reference data sets. Therefore, we added a parameter ‘ref.data’ to scMCA to import the average expression of each cell type for the reference data set, similar to its internal function to predict murine cell types. scPred [20] provides the option to call all models included in the caret package [32], and SVM with radial basis function kernel is called by default. Seurat implements cell type classification using its ClassifyCells function, which is an interface to randomForest package [33]. CaSTLe [34] uses XGBoost and requires logcounts of SingleCellExperiment objects as its data format. scID [35] first performs a feature selection step for each reference cell type through FindMarker function of Seurat and then deduces corresponding reference cell type membership of target cells employing a Fisher’s linear discriminant analysis classifier. AltAnalyze [36] is an integrated pipeline for analysis of scRNA-seq data sets and implements a sample classification using its LineageProfilerIterate.py script as a command line tool. It requires one or more gene models, namely gene lists as one of the input files. If not provided, it will return the intersection of expressed genes between reference and test data sets. The union set of genes of reference and test sets are adopted as gene list in this article. CellFishing [28] is similar to scmapc2c but uses locality-sensitive hashing to hash expression profiles into bit vectors. It then estimates cosine similarity between two cells from their Hamming distance. CellFishing is specifically compared to scmapc2c in its published article due to their similarities. In all tools, scmap, scPred and scID have the capability to predict certain cells as ‘unassigned’ when the similarity/probability/score is lower than a certain threshold or not returned by the model. In scmapc2c, the cell is also labeled as ‘unassigned’ if its nearest *k* neighbors are not from the same cell type. Main parameters and default values of tools in Table 1 are shown in Supplementary Table S6.

Some tools were excluded from this evaluation due to various reasons (Supplementary Table S5). For example, celaref and MetaNeighbor [37] require clustering of the test data prior to classification, and thus, their performances are partially dependent on the clustering. DistMap [38] is designed for cell classification of 3D gene expression. Moana [39] only provides a pretrained classifier for PBMC as of 31 December 2018.

### Strategies of performance evaluation

#### Construction of reference and test data pairs

To get a performance assessment of classification tools under different scenarios, we set up eight pairs of reference and test data sets, all generated from three sources discussed above (Table 2). The test pairs are designed to evaluate three levels of effects with increasing variability on the performance of tools: (1) The reference and test data sets were randomly selected from the same scRNA-seq data set (self-projection). Self-projection represents an ideal but unrealistic situation. (2) The reference and test data sets were from two different scRNA-seq experiments on the same platform, originated from the sample biological material. This scenario is to mimic the use case when a lab wants to increase their sample size based on a small-scale preliminary study. Batch effects between experiments could potentially affect the prediction accuracies. (3) The reference and test data sets were generated from the same tissue but were from different biological individuals using different platforms by different labs. In addition, the data sets were processed by different computational analysis pipelines. This is the most realistic setting and represents the most applicable use case, predicting cell types for any new scRNA-seq experiment based on existing public scRNA-seq data sets.

**Table 2:** Eight pairs of data sets used in the performance evaluation of all tools

Data sets	PBMC	Mix cell lines			Pancreas of human			
Reference	500*10	80% Mix4	Mix4	Mix3	Baron	Baron	Muraro	Muraro
Test	50*10	20% Mix4	Mix3	Mix4	Muraro	Xin	Baron	Xin
Situation	Self-projection		Projection from one data set to another from different experiments					

*Note.* See Methods section for original data set description.

For self-projection of mixed cell lines, we randomly split Mix4 data set into 80%:20% as the reference and test data sets, respectively. For self-projection of PBMC, 500 cells were randomly sampled from each data set of purified cell types without replacement and combined into the reference data set. Fifty cells were then selected randomly without replacement from remaining cells of each data set and combined into the test data set. To have a fair comparison for the tools with the ‘unassigned’ function, we also tested scenarios where test data set contains novel cell types not presented in the reference data set. In reality, although novel cell type discovery may not be a main objective of the scRNA-seq experiments, there is no guarantee all cell types in the test data set are included in the reference data set when using classification methods. Thus, we used Mix3 with three cell types as a reference to predict Mix4 with four cell types. Similarly, we used the Xin data set, which only has four cell types, to predict the Muraro data set, which has nine cell types. More importantly, all classification tools were investigated with default parameters or recommended parameters to ensure the fairness of evaluation. Performances of tools with default parameters reflect their robustness and applicability, which is an important criterion for researchers without enough bioinformatics expertise to determine whether to use it as an off-the-shelf solution.

### Batch effects on the classification performances

There are significant batch effects between Baron and Muraro data sets for four cell types, alpha, beta, gamma and delta (Supplementary Figure S2). Cells of these four cell types from Baron and Muraro data sets are extracted as a pair of reference and test data sets to assess the influence of batch effects on classifiers.

### Influence of numbers of reference cells on the performance

Reference cell numbers could potentially affect the performance of tools, as observed in other supervised classifier evaluations. Fifty, 100, 250, 500, 1000 and 2000 cells per PBMC cell type were selected randomly and combined to form reference data sets**.** We down-sampled the reference cells sequentially where a larger reference data set always contains all cells in a smaller reference data set. We used the same test data set with 50 cells per cell type in all predictions in this section.

### Performance stability

Different samplings could potentially have an effect on the results. In order to achieve unbiased evaluations and examine stabilities of all tools, we generated 100 sets of data pairs for both PBMC and Mix4 self-projections. For PBMC, 500 cells were selected as the reference data set and 50 cells as test for each cell type in each sampling. Mix4 data set was randomly divided into five equal groups for 100 times. For every division, one of the groups was chosen as the test data set, and the rest as the reference data set. Meanwhile, 10 times 10-fold cross validations (CVs) were used to see if it is susceptible to introduce bias due to the random determination of training and test sets for Mix4 and PBMC data sets.

### Effects of classifier parameters on performance

To understand the association between parameters and performances of tools, scmapc2c, scPred and scID were evaluated by tuning their parameters on PBMC data sets. In scmapc2c, we varied two parameters: the number of centroids and the threshold. Centroids are landmark points calculated by *k*-means clustering of cells, used to estimate the similarity between cells. The threshold is a cutoff to determine whether the evidence is strong enough to identify the cell type. For scPred, the threshold was fine-tuned, which is similar to the threshold parameter in scmapc2c. As for scID, one parameter named ‘contamination’, which affects the number of cells belonging to a certain cell type, is tuned.

### Class-imbalanced tests

Four PBMC cell types, CD19^+^ B cells, CD56^+^ natural killer (NK) cells, CD4^+^ helper T cells and CD4^+^ CD25^+^ regulatory T cells (Tregs), with various levels of similarities, in turn were selected to form class-imbalanced data sets. We compared the performance of class-imbalanced data sets with a control reference. In the class-imbalanced group, cells of four cell types were randomly selected without replacement and with the cell numbers of 10 000, 10, 10 000 and 10 to form the reference data set. And 50 cells were then selected randomly without replacement from remaining cells per cell type and combined into the test data set. In the control group, 500 cells of each of four PBMC cell types were randomly selected without replacement and combined to form the reference data set, and the test data set is similar to that of the class-imbalanced group and cells of it do not overlap with the reference data set.

### Evaluation metrics

We extracted the accuracy and recall from confusion matrices [40] using caret R package. They are defined as follows:

$$ \mathrm{Accuracy}=\frac{\mathrm{TP}+\mathrm{TN}}{\mathrm{TP}+\mathrm{TN}+\mathrm{FP}+\mathrm{FN}} $$

 

$$ \mathrm{Recall}/\mathrm{FPR}=\frac{\mathrm{TP}}{\mathrm{TP}+\mathrm{FN}} $$

ROC and AUC are used to assess the stability and robustness of classifiers. In this article, R packages pROC and multipleROC were used to calculate ROC curves with sensitivity and 1 − specificity as the axis.

$$ \mathrm{Sensitivity}=\frac{\mathrm{TP}}{\mathrm{TP}+\mathrm{FN}} $$

 

$$ \mathrm{Specificity}=\frac{\mathrm{TP}}{\mathrm{TP}+\mathrm{FN}} $$

In addition, Matthews correlation coefficient (MCC) takes into account both true and false positives and negatives, which is regarded as a balanced measure for class-imbalanced data sets [41, 42] and defined as the following formula:

$$ \mathrm{MMC}=\frac{\mathrm{TP}\ast \mathrm{TN}+\mathrm{FP}\ast \mathrm{FN}}{\sqrt{\left(\mathrm{TP}+\mathrm{FP}\right)\ast \left(\mathrm{TP}+\mathrm{FN}\right)\ast \left(\mathrm{TN}+\mathrm{FP}\right)\ast \left(\mathrm{TP}+\mathrm{TN}\right)}}$$

MCC is calculated using yardstick R package.

## Results

### Overall performance evaluation

**Figure 1:**
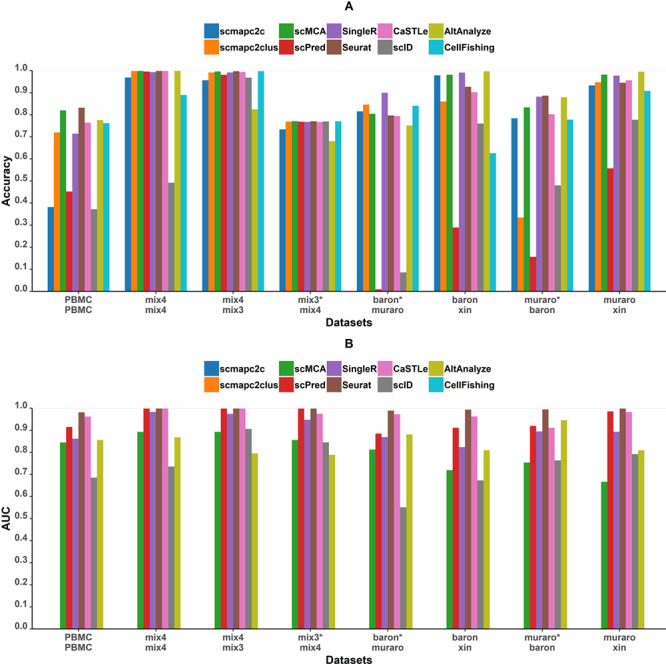
Performance of all tools on eight projections. The top and bottom names on the *x* axis represent the reference and test data sets, respectively. The pairs of data sets labeled with ‘*’ mean that test data sets contain novel cell types not included in the reference data set. **(A)** Accuracies of all tools across eight tests. **(B)** AUC of macro ROC curves of all tools except scmapc2c, scmapc2clus, CellFishing and AltAnalyze on eight pairs of data sets. These four tools do not output a probability or score value for each class and cannot be evaluated through ROC curves.

**Figure 2:**
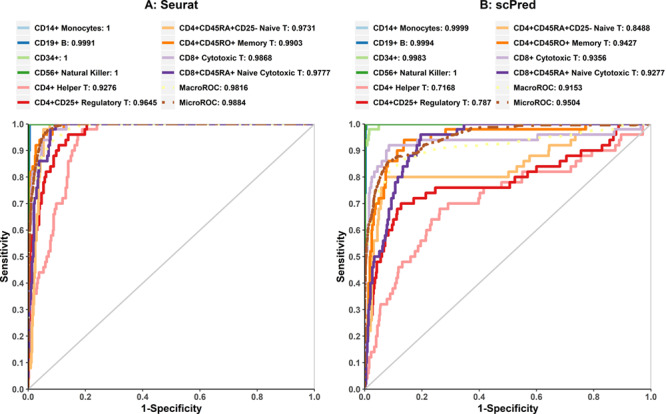
Analysis of cell type prediction accuracy of PBMC subtypes. ROC curves of Seurat **(A)** and scPred **(B)** on 10 cell types of the PBMC data set.

Figure 1 shows the classification accuracies and AUC of all tools for eight test cases. In term of accuracies within one data set, predictions of self-projection of mixed cell lines are nearly perfect, which is consistent with the expectation. scID performed worst in the self-projection of Mix4 with the lowest accuracy. In contrast, the accuracies obtained in the self-projection of PBMC are lower than that in the self-projection of mixed cell lines for all tools, which is due to the higher complexity of PBMC. To investigate the exact reason for overall low accuracies, we performed the ROC analysis to evaluate whether the classification abilities of tools are different for different cell types. For Seurat (Figure 2A), AUC values of cell types, except various subtypes of T cells, are equal or close to 1, indicating strong prediction powers. All T cells show lower AUC values, and CD4^+^ helper T cells and CD4^+^ CD25^+^ Tregs have the lowest AUC values. Similar results were observed for scPred (Figure 2B). Correlation analysis of PBMC cell types between reference and test data sets (Supplementary Figure S3A**)** indicated that T-cell types have very high correlations with each other. The two T subtypes with the lowest AUC values, CD4^+^ T helper and CD4^+^ Tregs, have a pairwise correlation of 0.99, even higher than the monocyte correlation between the reference and the test data sets. If similar T cells with CD4^+^ or CD8^+^ markers were considered as the same cell type (Supplementary Figure S4), especially when all T cells were considered as the same cell type, the accuracy of the predictions is higher than predicting T subtypes individually. Therefore, T-cell subtypes could be an intractable challenge to classify, while distinct cell types are easier to classify.

When projecting from Mix3 to Mix4, the accuracy is similar to that of the self-projection of Mix4, and all tools performed well. The projection from Mix4 to Mix3 showed nearly all accuracies are between 0.7 and 0.8 (Figure 1A). Since Mix4 has one cell type A431 not included in Mix3, tools without ‘unassigned’ function (see Methods section for details) classified A431 as other cell types in Mix4. After further analysis, we found that all accuracies not considering A431 are above 0.95 (data not shown). Most A431 cells are predicted to be 293T since A431 is most similar to 293T among the three cell types in Mix3 (Supplementary Figure S3B). In summary, all tools demonstrate good classification performances on mixed cell line data sets. It indicated that batch effects between experiments based on the same protocol are negligible for data sets with low complexity and have an insignificant impact on cell type predictions.

In projections among four pairs of human pancreas data sets, which are close to realistic situations and practical needs, tools’ performance varies. Accuracies in projections where Xin is the test set are overall higher than the other two, since Xin data set only contains four cell types and the difference among these four cell types is significant (Supplementary Figure S3C). The other two projections between Baron and Muraro data sets showed worse accuracies, in the range of 0.7–0.8, for all tools, except for even lower accuracies for scmapc2clus, scPred and scID. One reason is that Baron and Muraro data sets contain eight cell types in common with some cell types unique in each data set. Similar to projection from Mix4 to Mix3, those unique cell types could only be assigned with a wrong label or ‘unassigned’. In addition, mesenchymal cells only presented in Muraro and quiescent stellate cells only presented in Baron are highly related cell types, which are frequently predicted to be each other. It is worth noting that scmapc2clus performed worst compared with other groups when Muraro is the reference set. Through further investigation, we found that accuracy observably increased with the parameter ‘threshold’ decreasing (Supplementary Figure S5). It indicated that the ranks of similarities between query cells and reference cell types are correct, but some of the cells were predicted as unassigned due to an inappropriate threshold. scmapc2clus constructed a representative gene expression profile for each cell type using the median expression of each gene. Low cell numbers of certain cell types in Muraro might cause the cell type level gene expression profiles to be not representative. Hence, lower similarities arose between reference cell types in Muraro and their corresponding type of test cells. For the projection from Muraro to Baron, since cell numbers of common reference cell types are large enough, the performance of scmapc2clus improved.

Compared among the tools, the one with the best accuracy is different under various test strategies. Seurat, scMCA, SingleR and CaSTLe showed higher accuracies (greater than 0.7 across all data sets) than other tools on all eight pairs of data sets. In addition, their prediction accuracies are more stable across eight pairs of data sets. Therefore, these four tools possess better universal applicability for different data sets. scmap, including both scmapc2c and scmapc2clus, has an unstable performance with its default parameters. scmapc2c has the lowest accuracy in PBMC self-projection, and scmapc2clus has the worst performance in the pancreas group where Baron is the test set and Muraro is the reference set. scPred and scID have overall poor performance; scPred especially performed worse than other tools on all data sets except for mixed cell lines. This is because numerous cells were incorrectly predicted as ‘unassigned’ by these two tools, even though some cell types in the test data set are already included in the reference data set. Higher accuracy is achieved when calculations do not include unassigned labels. Meanwhile, AUC values (Figure 1B) of tools based on supervised learning are all higher than other tools. Even though scPred has lower overall accuracy, ROC curves suggested it has the potential to improve. SingleR performed worse than supervised learning tools but better than scID and scMCA in terms of AUC. AUC of scID is also low, consistent with its low accuracy. We conducted further analysis on scmapc2c, scPred and scID to evaluate the effects of the ‘unassigned’ function (see Tuning parameters section).

In summary, the performance of tools is partially dependent on the complexity of data sets, and there is no universally best tool under all circumstances. Results of projections between two mixed cell lines data sets and among three pancreas data sets suggest that batch effects, experimental protocols and biological variations have little impact on the prediction of well-separated cell types. In addition, all tools performed poorly for the PBMC data set with lower accuracies compared with other data sets, which is due to closely related cell types coexisting in PBMC. Hence, we used PBMC data sets in the following evaluation process. Moreover, an ensemble voting of tools on PBMC data set presented a slightly better accuracy (Supplementary Figure S6), which provide a new thought to correctly classify single cells with high similarity.

### Batch effects

**Figure 3:**
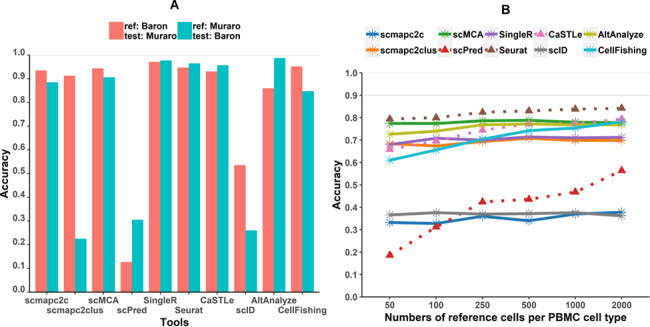
Accuracy of all tools with **batch effects and** different reference set size: **(A)** the influence of batch effects on the accuracy and **(B)** the accuracy during different sizes of reference data sets. Dash lines represent accuracy trends of machine-learning tools.

Batch effects are common between scRNA-seq experiments, especially when the data sets are from different experimental platforms or protocols. They are challenging for the combined analysis using clustering and batch effect correction methods have been proposed [43–45]. Therefore, classification tools that are not sensitive to batch effects could be beneficial. To test effects of batch effects on classifiers, cells of four cell types, alpha, beta, delta and gamma, from Baron and Muraro data sets are used to evaluate how batch effects influence classification performances. According to the accuracy of each tool (Figure 3A), scmapc2c, scCMA, SingleR, Seurat and CaSTLe are not sensitive to batch effects of data sets. A significant difference of accuracy between two test scenarios in scmapc2clus was due to fewer reference cells in Muraro than in Baron. As described above, the accuracy would improve by lowering the thresholds of scmapc2clus (Supplementary Figure S4). In total, scmapc2c, scCMA, SingleR, Seurat and CaSTLe are tolerant to batch effects between reference and test data sets.

### The effect of the number of reference cells on performance of tools

To investigate whether the number of reference cells makes an impact on the performance of classification tools, we created a series of PBMC reference data sets with various numbers of reference cells (see details in Methods section). Figure 3B displayed the accuracy profiles of all tools on reference data sets with different sample sizes. Accuracies of classification tools based on supervised learning, such as scPred, Seurat and CaSTLe, gradually increase with the increasing number of reference cells per PBMC cell type. In contrast, accuracies of the other tools barely change when changing the numbers of reference cells, especially the accuracies of scMCA and SingleR based on cell type-level distances. It suggested that when reference set size is small, it may be beneficial to use tools based on cluster-level similarities. In addition, we found that when the number of cells of each cell type is 500 or more, accuracies reach saturation for all classification tools on PBMC data sets. With increasing reference cells, time and memory consumption of tools would theoretically increase to various degrees. For tools based on cluster-level similarities, such as scmapc2clus and SingleR, the time and memory consumption would increase linearly with the increasing size of reference data when calculating mean/median expression profiles for each cell type. However, tools of supervised learning algorithms would take more time to train the model, such as scPred and Seurat, with a time complexity greater than *O*(${n}^2$) and CaSTLe with that greater than *O*($\log n$) ($n$ represents the number of training samples). Therefore, it indicates it might be appropriate to subsample each cell type to a sample size of 500 when more cells are available, in order to save computing resources and time while achieving the comparable accuracy.

### Bootstrapping test and 10-fold CVs of tools in self-projection

**Figure 4:**
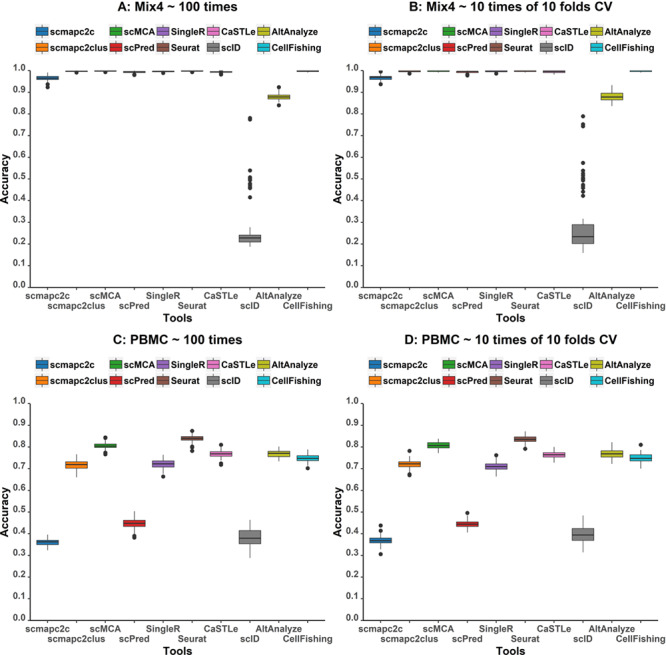
Box plots of accuracies of all tools tested on 100 Mix4 **(A)** and PBMC **(B)** samplings as well as 10 times 10-fold CVs tested on Mix4 and PBMC data sets.

In self-projection of Mix4, scID performed unstably on randomly sampled reference data, which may happen to other tools. We have demonstrated above that reference sample size of 500 cells per PBMC cell type is a good choice in terms of balancing between accuracy and efficiency. Hence, we performed 100 samplings of PBMC and Mix4 data sets and 10 times 10-fold CVs (see detailed information in Methods section) and evaluated their accuracies. Figure 4 displays the accuracy distribution of all tools on 100 samplings and 10 times 10-fold CVs. Variances of accuracies within one tool are very small except for scID. scID presented unstable accuracies even in Mix4 self-projection. Meanwhile, this indicated that bootstrapped samples have less impact on predictions for all tools when the number of reference cells per cell type is larger than 500 for PBMC.

### Tuning parameters

Three tools, scmapc2c, scPred and scID, performed much worse than other tools in the bootstrapping test and the training sample size test, accuracies of which are less than 0.5 (Figures 3B and 4**)**. ‘Unassigned’ labels occur when the tools decide cells are not close enough to any cell type in the reference data set or when a cell cannot be classified with enough confidence, which probably happens on cells of novel cell types not existing in the reference data set. Hence, ‘unassigned’ function could be critical for catching novel cell types not presented in the reference data set. In this study, we regarded predicted ‘unassigned’ labels that are truly novel cell types not included in the reference data set as true ‘unassigned’, and else as mistaken ‘unassigned’. Moreover, accuracy and the number of ‘unassigned’ labels rely on parameters of those tools, as shown above for scmapc2clus (Supplementary Figure S4). Therefore, we tested the performances of these tools with variable parameters (see details in Methods section) on the same data set to see if the true ‘unassigned’ rate would be increased and the mistaken ‘unassigned’ labels decreased. We expected to not only improve the accuracy of identifying cells of cell types existing in the reference data set but also accurately identify cells of cell types not included in the reference data set—decreasing the ratio of mistaken ‘unassigned’.

**Figure 5:**
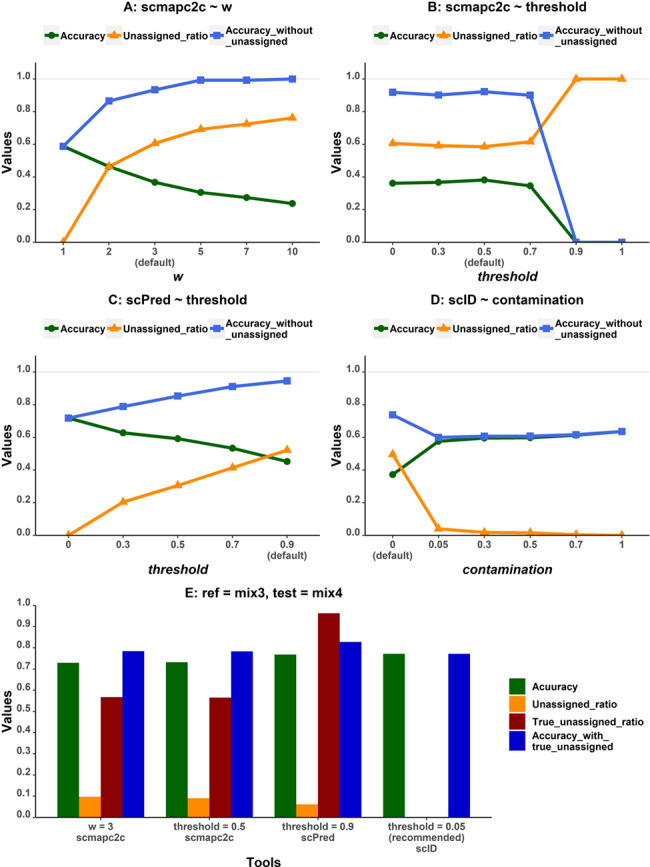
Performance of tools with the ‘unassigned’ function with various parameter choices: **(A)** parameter *w* of scmapc2c, **(B)** parameter threshold of scmapc2c, **(C)** parameter threshold of scPred and **(D)** parameter contamination of scID. Accuracy_without_unassigned means the accuracy calculated without considering ‘unassigned’ labels. Unassigned_ratio means the ratio of ‘unassigned’ labels in all predicted labels.

For scmapc2c, with the decreasing value of *w* in scmapc2c, which defines the number of nearest neighbors, accuracies get higher and the ratio of ‘unassigned’ labels to the total cell types in prediction gets lower (Figure 5A). It indicates that parameter *w* has a great impact on the prediction of scmapc2c by varying the number of ‘unassigned’ cells. Whereas with increasing similarity threshold (Figure 5B), the accuracy starts to decrease dramatically from the point where the threshold equals 0.5, the default value in scmap [19]. For scPred, a default threshold of 0.9 defines the minimum probability that one cell is predicted as a certain cell type, instead of ‘unassigned’. Figure 5C indicated that, with the threshold increasing, the accuracy declined and the unassigned ratio increased. As for scID (Figure 5D), which fits a mixture of two Gaussian distributions in a Fisher’s linear discriminant analysis classifier, the parameter ‘contamination’ represents the percentage of cells that located in the overlapping area between the population distributions of a certain cell type and others. With the increase of the value of ‘contamination’, the accuracy went up and the unassigned ratio went down. The accuracy tends to be stable when ‘contamination’ is larger than 0.05.

**Figure 6:**
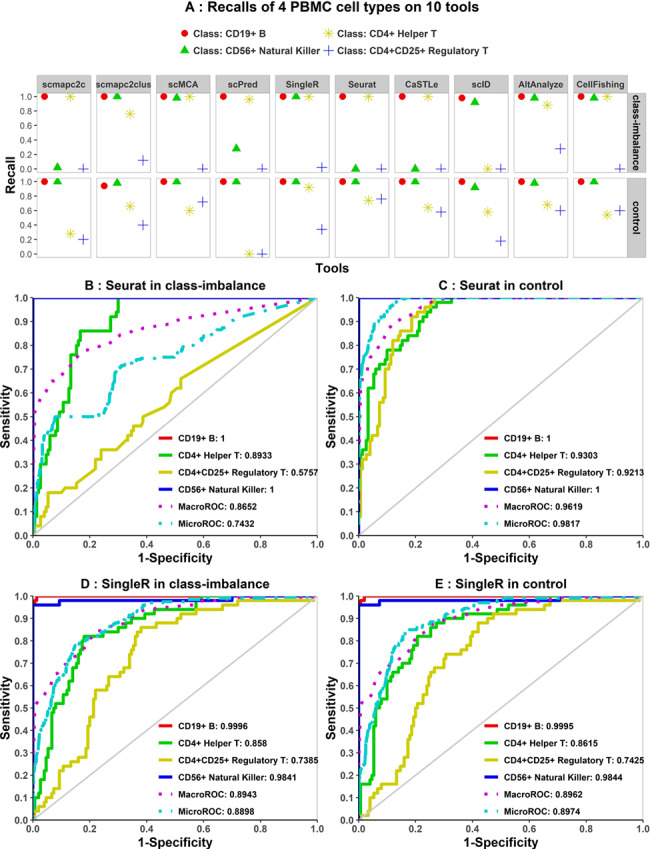
Recalls and ROC curves of four PBMC cell types in class-imbalanced and control group. **(A)** Recalls of four PBMC cell types in the class-imbalanced and control group. **(B and C)** ROC curves of four cell types in the class-imbalanced and control group in Seurat. **(D and E)** ROC curves of four cell types in two groups in SingleR. ROC curves indicated poorer robustness of Seurat on the class-imbalanced group than SingleR.

**Figure 7:**
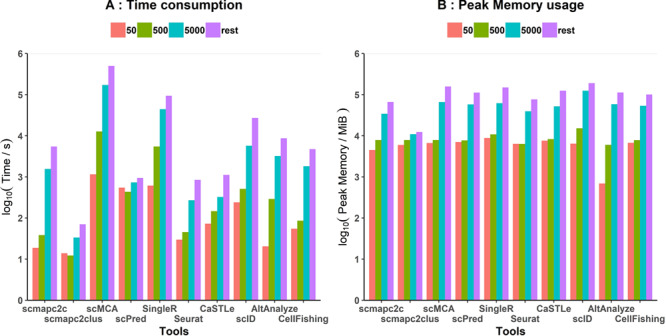
Time and memory consumption of all tools. **(A)** Time consumption and **(B)** peak memory usage of all classifiers, taking their logs base 10, with increasing test cell numbers. Nine PBMC cell types included, except CD14^+^ monocytes (due to its not enough cell number). Fifty, 500 and 5000 represented test cells per PBMC cell type were contained in the test data set. The ‘rest’ represented the total rest of cells apart from reference cells per PBMC cell type, about a total of 90,000 cells.

We defined a metric called unassigned ratio, as the ratio of cells with the ‘unassigned’ label to all target cells. Since the pair of PBMC data sets has the same cell types, all ‘unassigned’ cells are mistaken ‘unassigned’ cells. Mistaken ‘unassigned’ cells may not be considered as incorrectly predicted but are cells that need further analysis to define their cell types. Therefore, we also calculated the prediction accuracies without the ‘unassigned’ labels. The accuracies without ‘unassigned’ labels are higher than the accuracies with ‘unassigned’ labels for almost all tools (Figure 5), which indicated the ability of these tools to maximize the predictive accuracy for cells with definitive cell type labels. On the other hand, it is not desirable to have the ratio of mistaken ‘unassigned’ labels too high, which might require too much work downstream. In conclusion, after weighing these three metrics on the same reference and test data sets of PBMC, scmapc2c performed best with *w* equal to 2 or 3 and threshold between 0 and 0.7, scPred achieved better performance with the threshold between 0.5 and 0.7 and scID worked better with ‘contamination’ equal to 0.05. However, the accuracies of cells with definitive cell type assignment of these three tools are not significantly higher than the accuracies of other tools (Figures 1A and 5), unless more than half of the cells are deemed as ‘unassigned’.

To test the capability of these three tools to catch novel cell types not included in the reference data set, we conducted the same analysis on mixed cell lines data sets, with Mix3 as reference and Mix4 as test. A431 is the new cell type not in Mix3 but taking 22.84% proportion in Mix4. We found these tools mostly predicted A431 cells as wrong cell types, instead of ‘unassigned’ (Figure 5E). The proportion of true ‘unassigned’ cells in all ‘unassigned’ cells, defined as the true unassigned ratio, is greater than 0.9 in scPred, which means that scPred is the most capable to catch new cell types. In summary, it remains challenging for these tools to precisely catch new cell types, even for well-separated cell types. At the same time, this functionality brings noise into the prediction of data sets without novel cell types.

### The class-imbalanced test on tools

It is very common that multiple cell types with uneven proportions exist in the same scRNA-seq data set. The performance for such cases would reveal the robustness of classification tools. In this study, four PBMC cell types are selected (i.e. CD19^+^ B cells, CD56^+^ NK cells, CD4^+^ helper T cells and CD4 ^+^ CD25^+^ Tregs). The former two are remarkably different from each other, and the latter two are similar to each other (Supplementary Figure S3A). Cells of four cell types were randomly sampled separately and combined into two groups: the class-imbalanced group and the control group. The control group has four cell types taking equal proportions (see Methods section).

Recalls of four PBMC cell types representing the predictive accuracy of each cell type were calculated for both groups (Figure 6A). In the control group, all tools performed worse with lower recall rates on similar PBMC cell types, CD4^+^ helper T cells and CD4^+^ CD25^+^ Tregs, than the other two, which is in line with our expectation and previous results. However, in the class-imbalanced group, due to fewer reference cells of CD56^+^ NK cells, tools based on supervised learning, such as scPred, Seurat and CaSTLe, predicted worse than other tools except for scmapc2c, even if NK cells are significantly different from other PBMC cells. Compared with the control group, due to more CD4^+^ helper T cells added into the reference set of class-imbalanced group, more CD4^+^ helper T cells of the test set were correctly predicted by most tools, even though they are similar to CD4^+^ CD25^+^ Tregs and difficult to accurately predict in theory. Meanwhile, worse predictions of Tregs and NK cells in scmapc2c indicate that scmapc2c is sensitive to the number of reference cells per cell type. scID also shows poor robustness, which may be due to multiple factors, for instance, different samplings and similarity between cell types.

ROC curves (Figure 6B–E and Supplementary Figure S7) displayed that AUC values per cell type in Seurat were not as stable as those in SingleR between two groups. It also implied that the best performance of Seurat in the control group did not translate in extremely imbalanced data sets, whereas SingleR is relatively robust.

As reported by MCC (Supplementary Figure S8), Seurat and CaSTLe showed lower performances in the class-imbalanced group versus the balanced group, whereas others had nearly equal MCC values between the two groups. Owing to incorrectly predicted ‘unassigned’ labels (Figure 6A) in both balanced and imbalanced test cases, MCC values are low for both in scPred. In summary, different metrics imply similar conclusions, and we showed that tools based on supervised learning are less robust than the tools based on cluster-level similarities for class-imbalanced data sets.

### Running time and memory usage

Strictly speaking, numbers of reference and test cells both have impacts on the scalability of tools. We discussed in a previous section the impact of increasing reference cells on the time and memory consumption in theory and showed a reference data set larger than 500 per cell type might not be beneficial for achieving better classification accuracy (Figure 3B). Therefore, we only focus on the impact of increasing test cells on time and memory consumption in this section. We used a series of PBMC data sets to compare the program running time and memory usage of tools (Figure 7). All analyses were performed on the same device with two processors, Intel(R) Xeon(R) CPU E5-2650 v4 (2.20GHz) and 192GB of RAM (DDR4), as well as Ubuntu 16.04.6 LTS system.

In theory, since tools evaluated in this study would predict labels independently for each test cell, running time and memory usage should increase linearly with the increasing test cells. According to the results, run time and peak memory usage of tools roughly increased linearly with increasing test cell numbers (Figure 7), but accuracy barely changed (Supplementary Figure S9A). Specifically, scmap (scmapc2clus and scmapc2c) package ran significantly faster than other tools. Seurat, CaSTLe, AltAnalyze and CellFishing were also relatively fast. Whereas SingleR and scMCA took more time, running about 26 and 139 hours, respectively, when predicting approximately 90,000 cells. In terms of memory usage, all tools consumed similar peak memory with increasing test cells. scID, scMCA and SingleR consumed larger memories, especially scID with about 186G peak memory usage when predicting approximately 90,000 cells. Seurat, scmapc2c and scmapc2clus used less memory, especially scmapc2clus, maintaining small memory usage as test cells increase. Comprehensively, Seurat is a relatively ideal classification tool with higher accuracy, good scalability, faster running speed and smaller peak memory usage. In addition, similar tests were performed on an scRNA-seq data set of the mouse gut endoderm with >100,000 cells [46]. Consistent with the previous results, Seurat worked better in terms of high performance and low resource requirement compared to other tools (Supplementary Figure S9B–D).

## Discussion

In this study, we conducted a comprehensive evaluation of the performance of nine software tools for single-cell classification analysis. Using three sources of scRNA-seq data sets with different complexities, mixed cell lines as a golden standard, PBMC as a complex system and public scRNA-seq data sets of human pancreas as a near realistic situation, we evaluated tools for classifying single-cell labels by various evaluation metrics.

In projections of mixed cell lines and human pancreas data sets, most of the tools precisely predicted test cells with cell types existing in both reference and test data sets. However, for catching new cell types, such as A431, not included in Mix3, tools with the unassigned function, scmapc2c, scPred and scID, merely caught a small portion of the new cell type. In projections of PBMC, all tools performed worse than other test conditions due to the existence of several similar T-cell types. Most tools wrongly predicted similar T cells, and tools with the unassigned function predicted most of similar T cells as ‘unassigned’. By combining T subtypes into one label, the accuracies of tools went up. For tools with the unassigned function, most of cells with definitive cell type labels are correctly predicted and credible, especially in predictions of scPred. However, these tools with ‘unassigned’ function do not significantly outperform other tools even if considering accuracies without unassigned and provide no further solution to ‘unassigned’ labels. Here, we provide several potential strategies for unassigned cells. We would classify query single cells based on the reference data set using existing supervised classifiers with ‘unassigned’ function. To address query cells predicted as ‘unassigned’, one possibility is unsupervised analysis, to cluster unassigned cells and identify differentially expressed genes (DE genes) for each cluster. Then, DE genes per cluster could be compared with those of known cell types in the reference data set. Clusters with similar DE genes to reference cell types may be defined as corresponding cell types, and clusters with DE genes significantly different to the reference cell types could be defined as a new cell population. We could also cluster all cells in the query data set. By studying the proportion of unassigned cells in every cluster, we could distinguish between new cell populations and known cell types with noise. Certainly, these strategies would require further validations to better define labels of unassigned cells and discover new cell populations.

All tools studied in this article first conduct feature selection before classifying single cells, except for scMCA and AltAnalyze. And some of these tools also provide custom feature selection options. Feature selection is a general and critical step in the classification field, which could largely affect the performance of classifiers and the runtime. scMCA does not execute feature selection and directly calculates the correlation between test and reference expression profiles. Hence, scMCA consumed most runtime among all tools. Due to the complexity of feature selection, we did not test its effects on the tool performance in this article. With more and more tools developed in this field, feature selection may be an essential component in the future performance optimization.

Our evaluation suggests researchers knowing their reference data well before implementing cell type classifiers. By analyzing the similarity between cell types in the reference data set, we could set lower expectations for prediction accuracies of highly related cell types. Similar subtypes could be combined to increase the accuracy. Seurat, SingleR and CaSTLe are the best tools, while an ensemble voting of them presented a slightly better accuracy. When the cell number is small or cell types are extremely imbalanced in the reference, SingleR is the best choice, based on the cluster-level similarities. If there might be novel cell types in the target cells, a combined strategy of using scPred with the ‘unassigned’ function, a well-performed tool without the function such as SingleR, and the clustering analysis might achieve a better outcome.

## Conclusion

In summary, more and more tools are developed for identifying cell types of single cells, yet researchers still face many challenges. We evaluated the functionality and efficiency of all available single-cell classification tools in this study. As of now, there is no tool that can perfectly and completely solve all problems. Based on the accuracy, ROC and other evaluation metrics, we demonstrated that Seurat, SingleR and CaSTLe outperformed the rest of tools. Although scMCA also performed relatively better, it is time-consuming. Tools built on cluster-level similarities are more robust than tools based on supervised learning for nonideal reference data. To use a reference data set consisting of several similar cell types, researchers could merge them into one super type for better prediction accuracy, or caution should be taken for potential mixed-up cell labels. Although novel cell types and closely related cell types are still very intractable problems, the results of our tests suggest that it is applicable to incorporate classification into the single-cell analysis workflows. In the future, feature selection and enhancement of novel cell prediction are worth further exploration to improve the accuracy and functionality of single-cell classification tools.

## Availability of data sets

scRNA-seq data sets of mix cell lines are available at the Gene Expression Omnibus at the accession number GSE128982. PBMC data sets can be downloaded from 10X Genomics official website (https://support.10xgenomics.com/single-cell-gene-expression/data sets) [9]. As for the human pancreas scRNA-seq data sets, they are converted into Bioconductor SingleCellExperiment class objects with cell type annotations and available on https://hemberg-lab.github.io/scRNA.seq.data sets [15]. The mouse gut endoderm scRNA-seq data set was downloaded from https://endoderm-explorer.com [46].

## Author Contributions

Xinlei Zhao performed the data analysis and data interpretation and wrote and edited the article. Shuang Wu performed the single-cell RNA-seq experiments of mixed cell lines. Nan Fang provided resources and supervised the work. Xiao Sun provided resources, edited the article and supervised the work. Jue Fan conceived the project, interpreted the data and wrote and edited the article.

## Supplementary Material

Figure_S1_bbz096

Figure_S2_bbz096

Figure_S3_bbz096

Figure_S4_bbz096

Figure_S5_bbz096

Figure_S6_bbz096

Figure_S7_bbz096

Figure_S8_bbz096

Figure_S9_bbz096

Table_S1_bbz096

Table_S2_bbz096

Table_S3_bbz096

Table_S4_bbz096

Table_S5_bbz096

Table_S6_bbz096

## Notes

Key Points

- Performance of classification tools is partially dependent on data sets.
- Seurat, SingleR and CaSTLe have overall better performance, and an ensemble voting of all tools increases the performance.
- SingleR performs best when the reference cell number is small or the cell types are imbalanced.
- Novel cell types are challenging to identify, and the function needs further improvement.

## Notes

**Xinlei Zhao** is a master’s student at State Key Laboratory of Bioelectronics, School of Biological Science and Medical Engineering, Southeast University, under the supervision of Professor Xiao Sun. He is working on collaborative projects with Singleron Biotechnologies in the area of single-cell analysis.

## Notes

**Shuang Wu** is a former employee at Singleron Biotechnologies.

## Notes

**Nan Fang** is the founder of Singleron Biotechnologies. Dr. Fang has over 10 years of experience in product development, commercialization, team management and corporate strategy in world-leading biotechnology companies in Germany, the United States and China.

## Notes

**Xiao Sun** is a professor in bioinformatics, working jointly at State Key Laboratory of Bioelectronics, School of Biological Science and Medical Engineering, Southeast University. He mainly engages in bioinformatics research, focusing on high-throughput DNA sequencing data mining and epigenetic information analysis.

## Notes

**Jue Fan** is the Vice President of Bioinformatics at Singleron Biotechnologies. Dr. Fan is mostly interested in applying statistical methods and machine learning techniques in the next generation sequencing data analysis, especially in single-cell analysis.
