# Supplementary figures and images for: Evaluation of single-cell classifiers for single-cell RNA sequencing data sets
Source: Brief Bioinform. 2019 Oct 23;21(5):1581–95. doi: 10.1093/bib/bbz096 (PMC7947964; doi:10.1093/bib/bbz096)

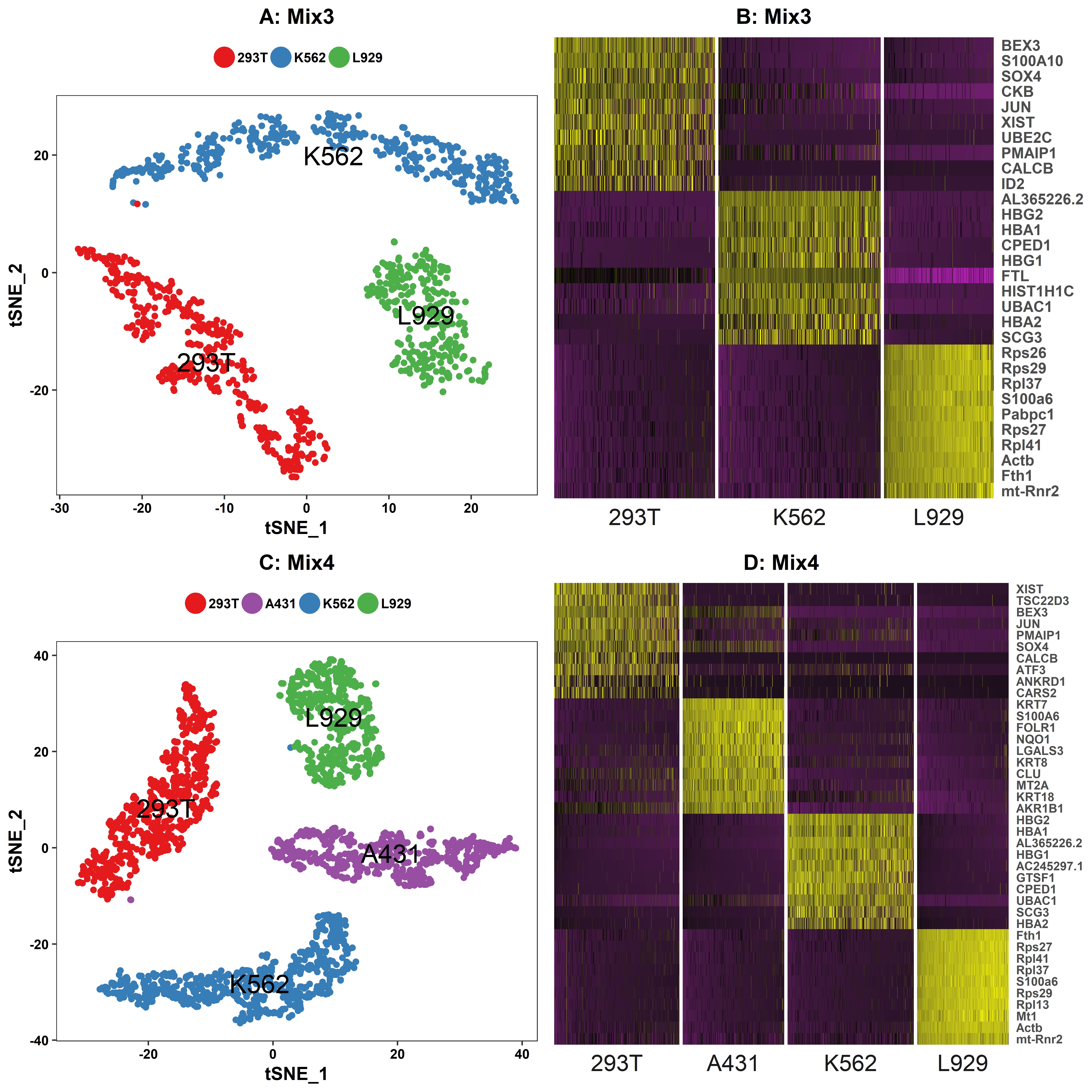

Supplement: Figure_S1_bbz096 [file figure_s1_bbz096.jpeg]

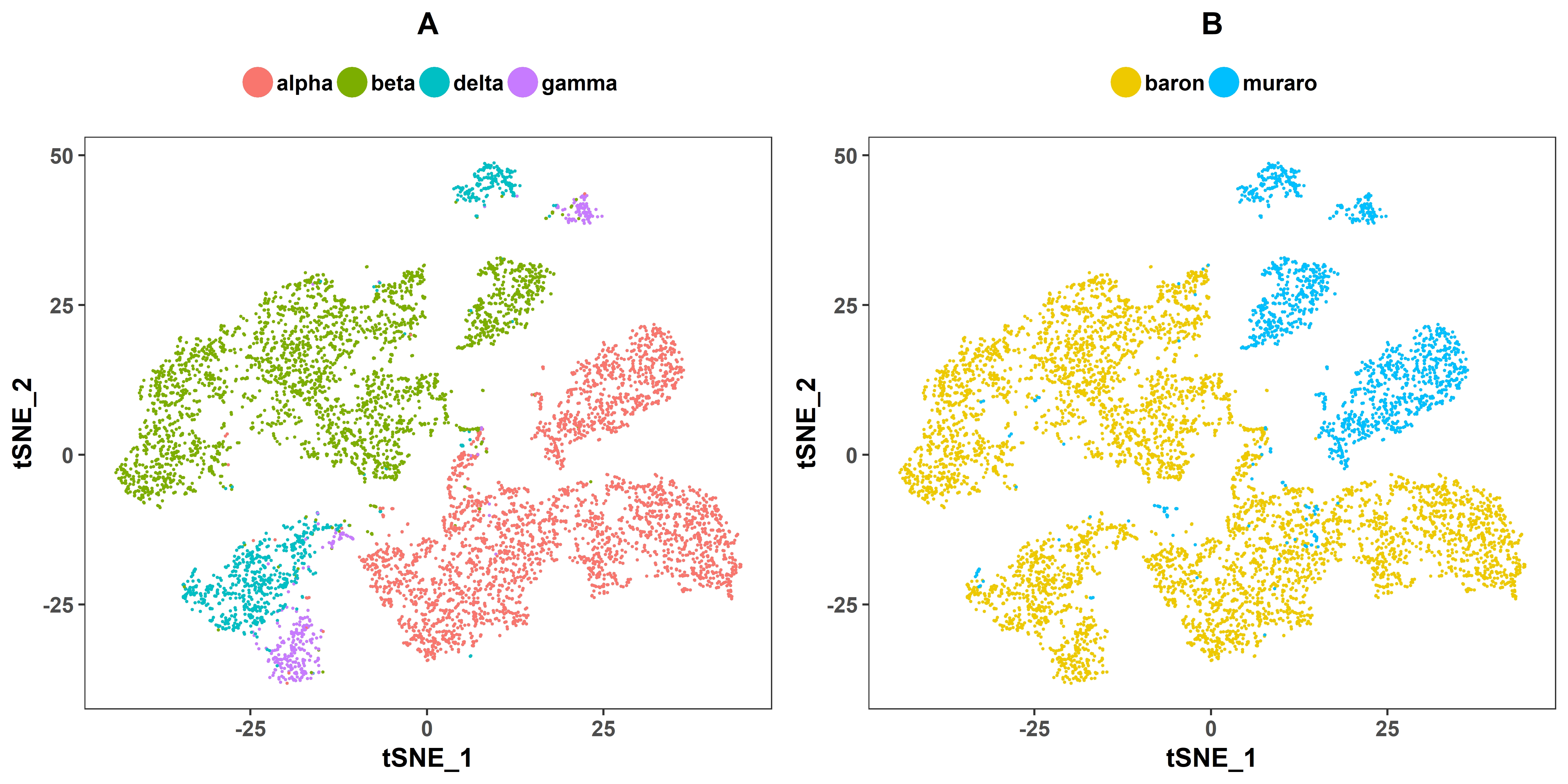

Supplement: Figure_S2_bbz096 [file figure_s2_bbz096.jpeg]

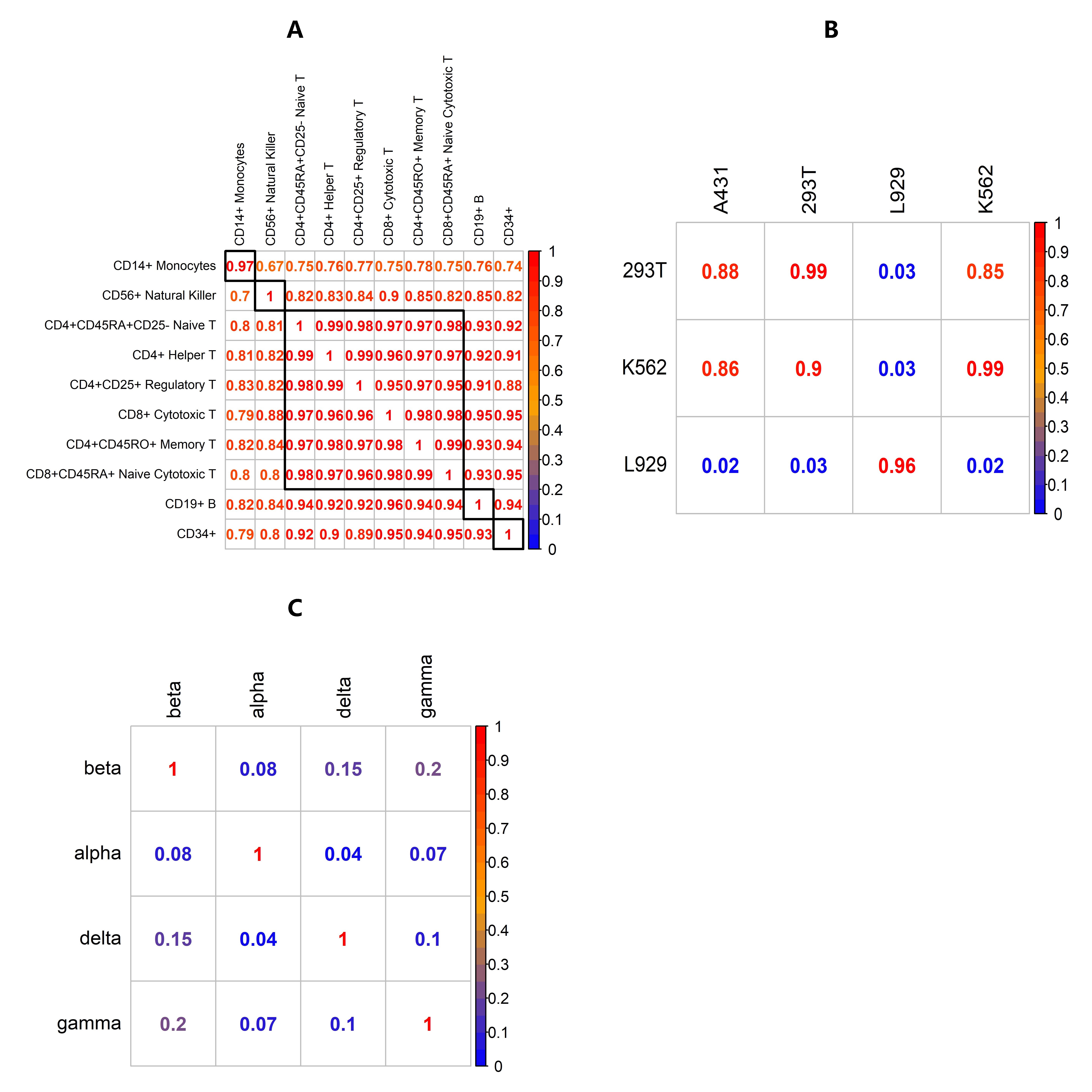

Supplement: Figure_S3_bbz096 [file figure_s3_bbz096.jpeg]

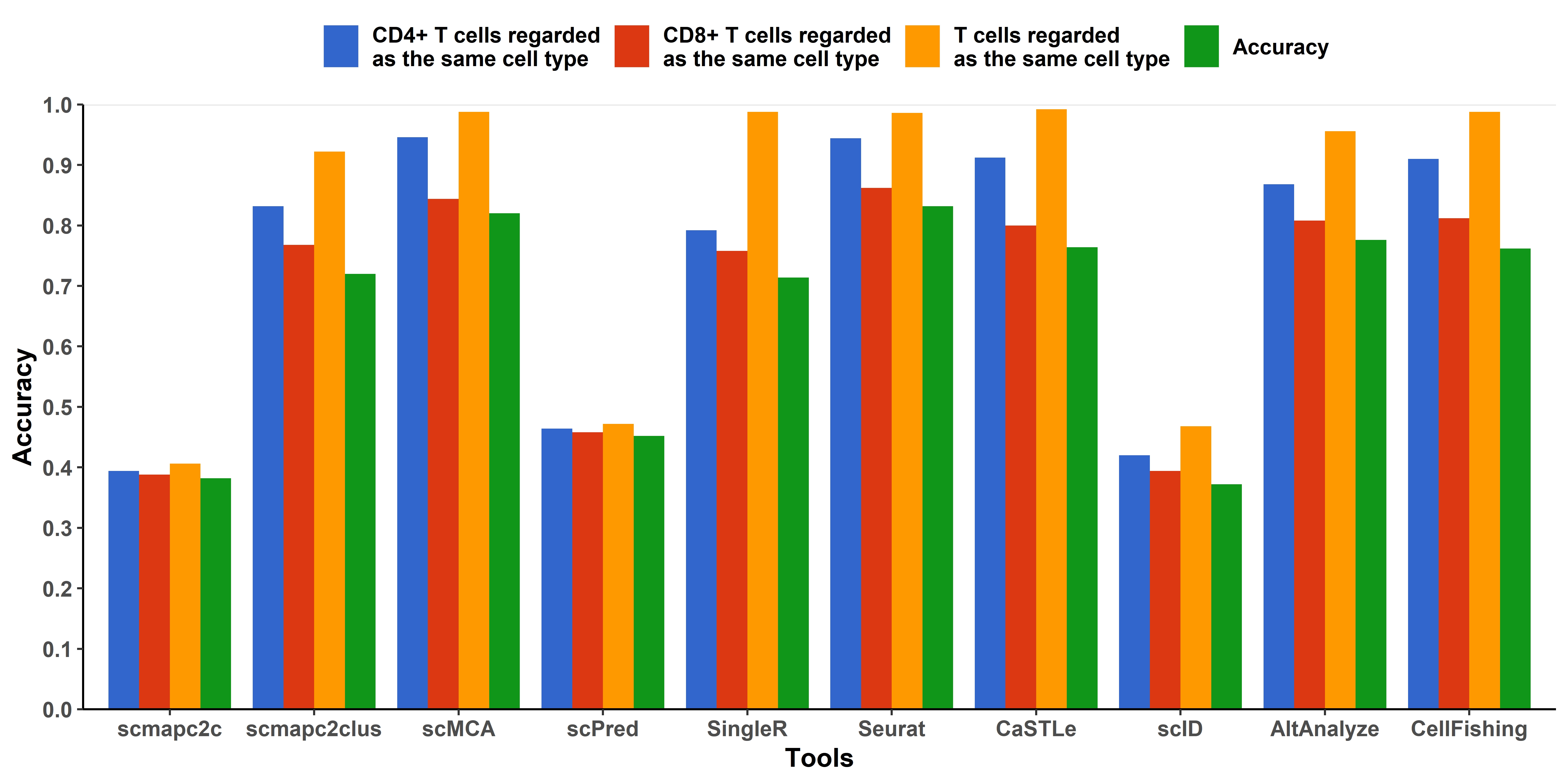

Supplement: Figure_S4_bbz096 [file figure_s4_bbz096.jpeg]

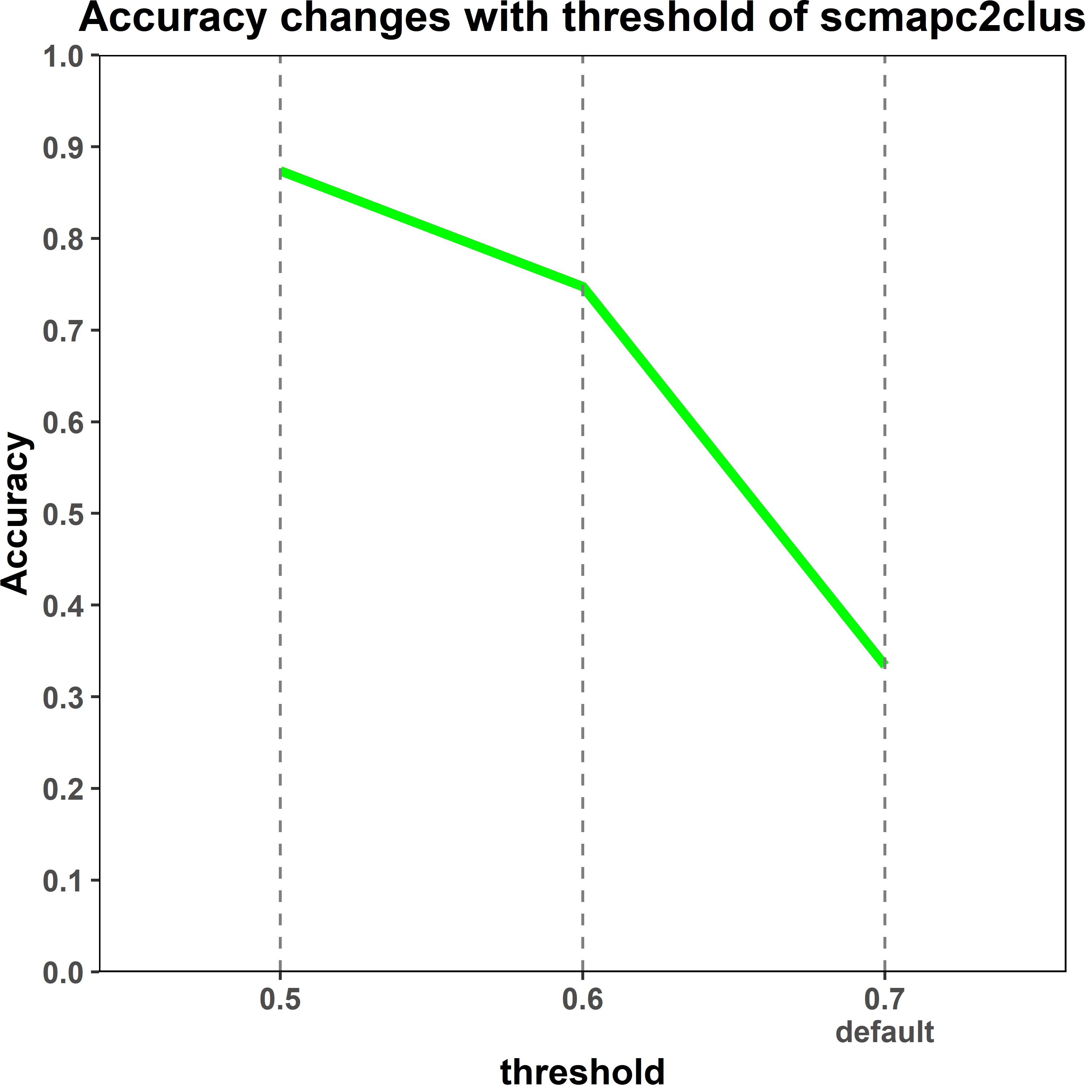

Supplement: Figure_S5_bbz096 [file figure_s5_bbz096.jpeg]

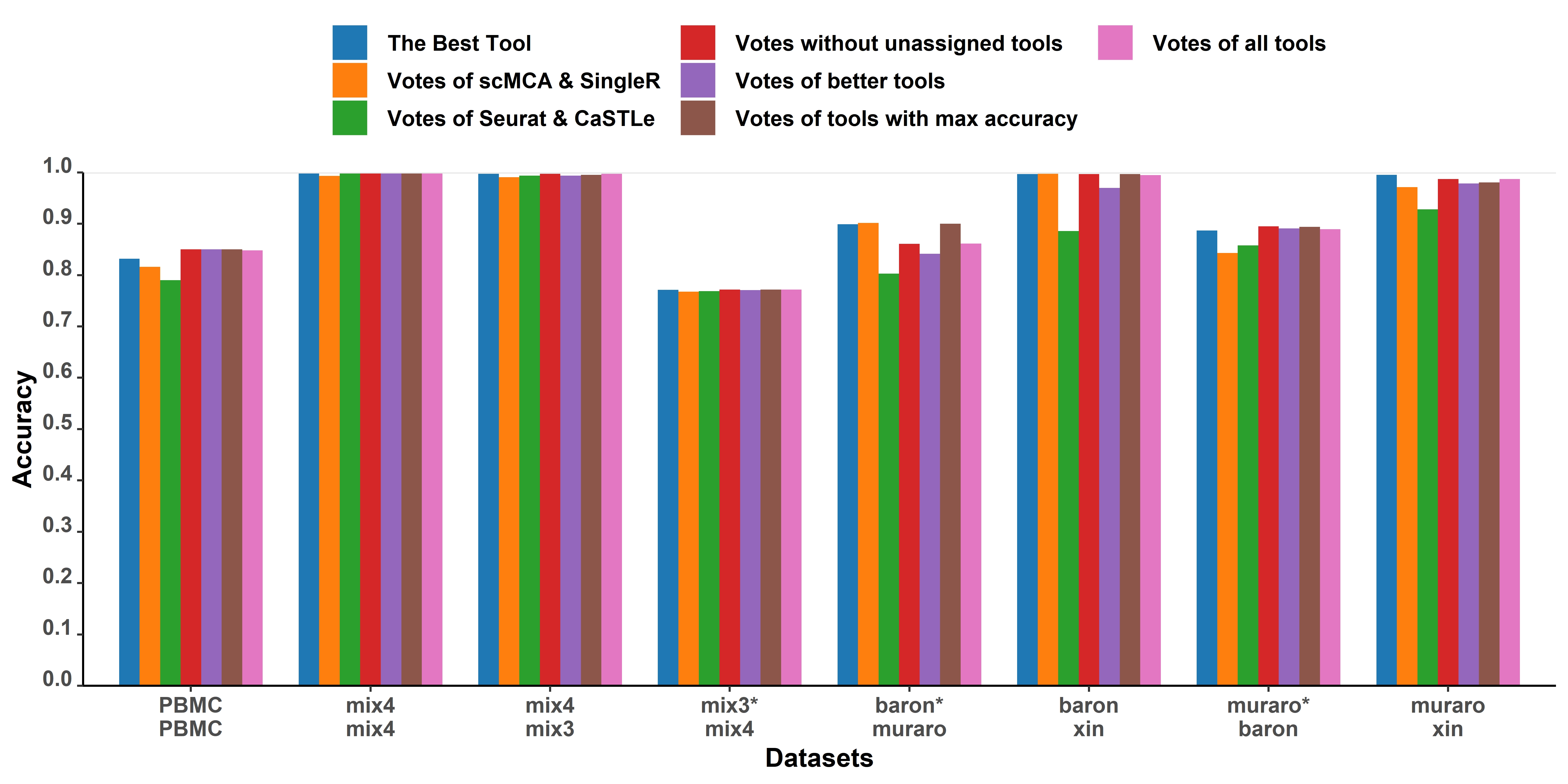

Supplement: Figure_S6_bbz096 [file figure_s6_bbz096.jpeg]

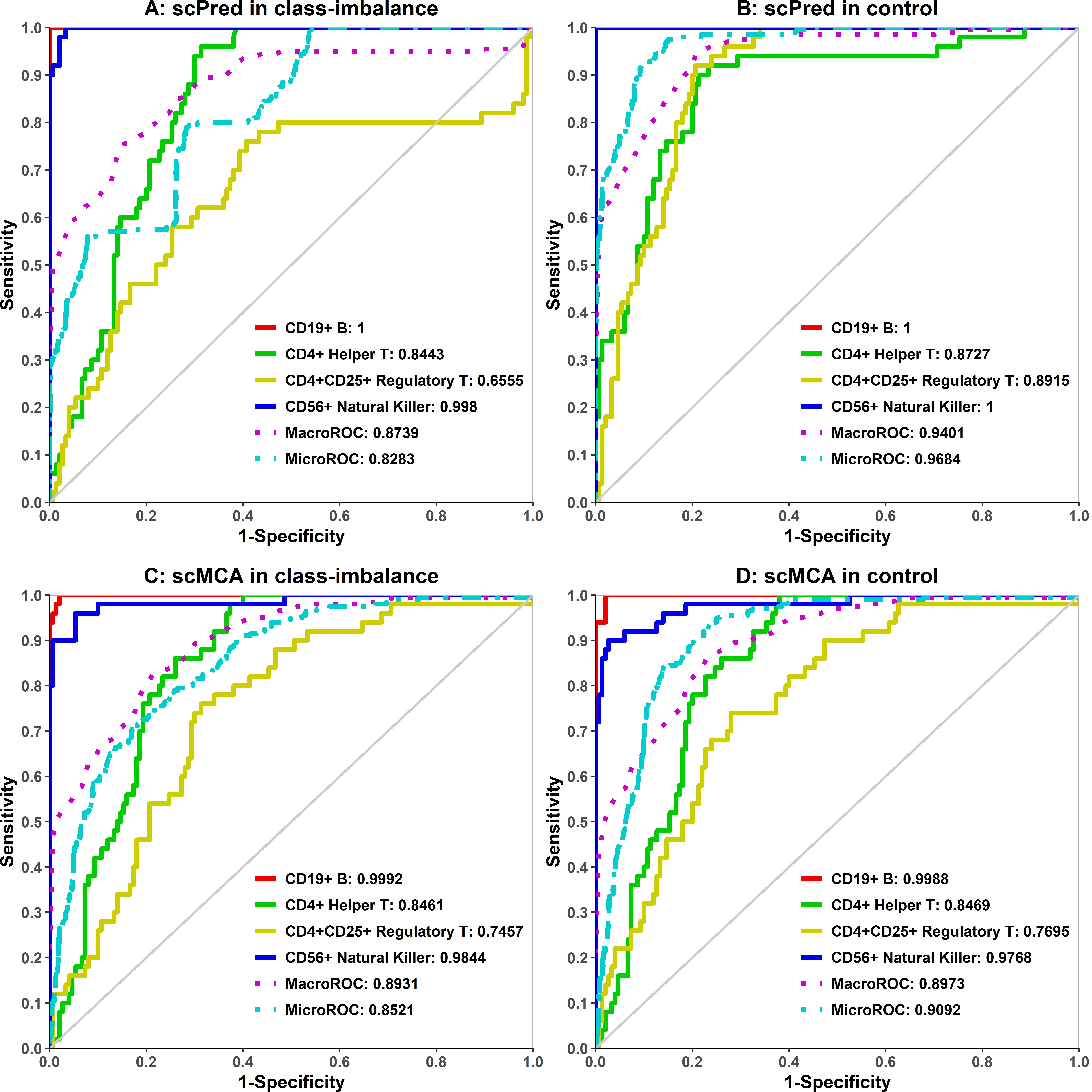

Supplement: Figure_S7_bbz096 [file figure_s7_bbz096.jpeg]

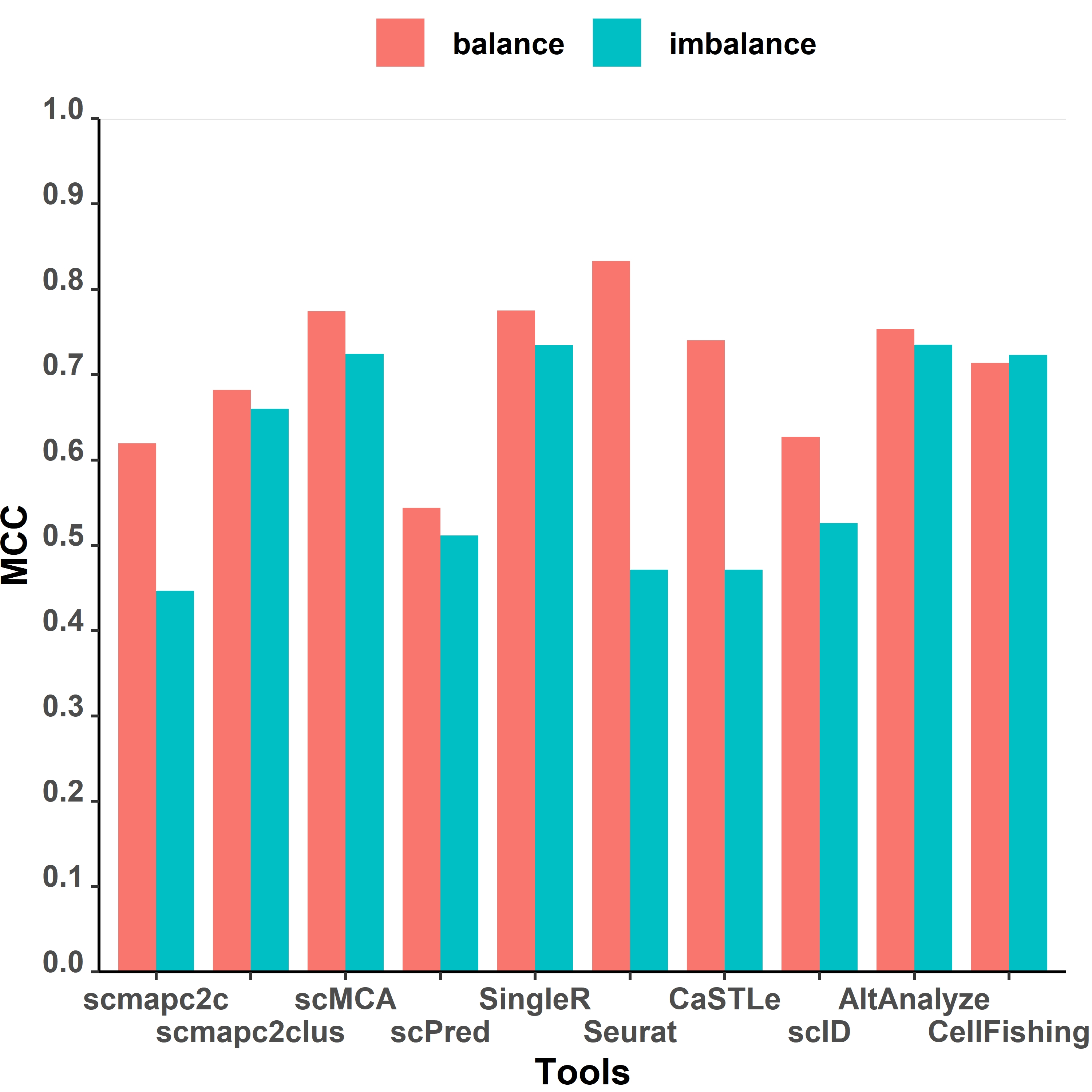

Supplement: Figure_S8_bbz096 [file figure_s8_bbz096.jpeg]

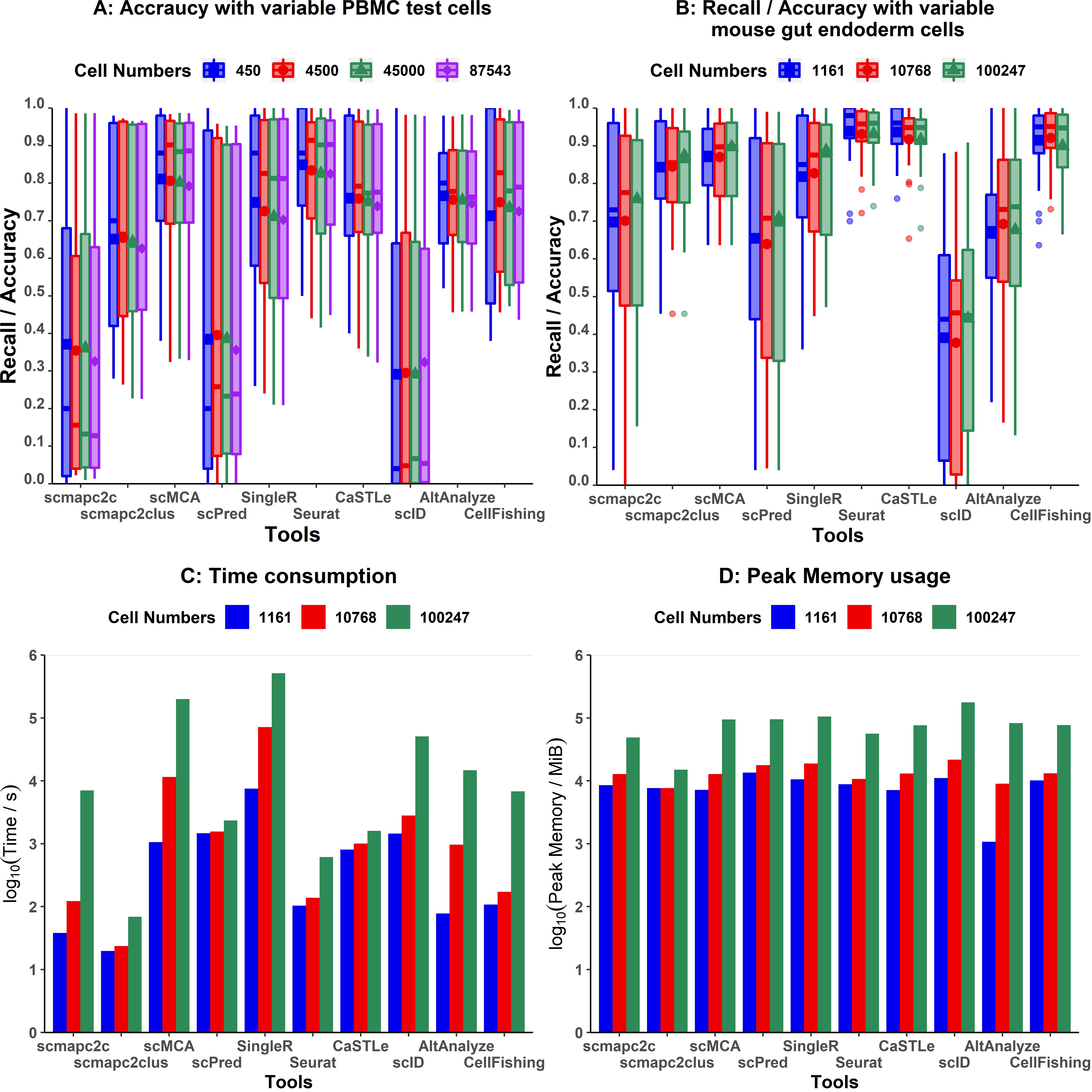

Supplement: Figure_S9_bbz096 [file figure_s9_bbz096.jpeg]
