# Supplementary material for: Evaluation of single-cell classifiers for single-cell RNA sequencing data sets
Source: Brief Bioinform. 2019 Oct 23;21(5):1581–95. doi: 10.1093/bib/bbz096 (PMC7947964; doi:10.1093/bib/bbz096)
Supplement: Table_S1_bbz096 [file table_s1_bbz096.docx]

| Experiments | Exp1 | | | Exp2 | | | |
| --- | --- | --- | --- | --- | --- | --- | --- |
| Cell Lines | 293T | K562 | L929 | A431 | 293T | K562 | L929 |
| Cell counts | 409 | 413 | 279 | 506 | 624 | 629 | 456 |
| Dataset Name | Mix3 | | | Mix4 | | | |

**Table S1.** Cell numbers of each cell type in Mix3 and Mix4 datasets.
