# Supplementary material for: Evaluation of single-cell classifiers for single-cell RNA sequencing data sets
Source: Brief Bioinform. 2019 Oct 23;21(5):1581–95. doi: 10.1093/bib/bbz096 (PMC7947964; doi:10.1093/bib/bbz096)
Supplement: Table_S2_bbz096 [file table_s2_bbz096.docx]

| **Cell Types** | **Cells counts** |
| --- | --- |
| CD14+ Monocytes | 2612 |
| CD19+ B Cells | 10085 |
| CD34+ Cells | 9232 |
| CD4+ Helper T Cells | 11213 |
| CD4+CD25+ Regulatory T Cells | 10263 |
| CD4+CD45RA+CD25- Naive T cells | 10479 |
| CD4+CD45RO+ Memory T Cells | 10224 |
| CD56+ Natural Killer Cells | 8385 |
| CD8+ Cytotoxic T cells | 10209 |
| CD8+CD45RA+ Naive Cytotoxic T Cells | 11953 |

**Table S2.** PBMC cell types and cell numbers per cell type.
