# Supplementary material for: Evaluation of single-cell classifiers for single-cell RNA sequencing data sets
Source: Brief Bioinform. 2019 Oct 23;21(5):1581–95. doi: 10.1093/bib/bbz096 (PMC7947964; doi:10.1093/bib/bbz096)
Supplement: Table_S3_bbz096 [file table_s3_bbz096.docx]

| Datasets | Accession | Protocol | Number of Cell Types | Cells | Genes |
| --- | --- | --- | --- | --- | --- |
| [Baron](http://dx.doi.org/10.1016/j.cels.2016.08.011) | [GSE84133](https://www.ncbi.nlm.nih.gov/geo/query/acc.cgi?acc=GSE84133) | [inDrop](http://dx.doi.org/10.1016/j.cell.2015.04.044) | 14 | 8569 | 20215 |
| [Muraro](http://dx.doi.org/10.1016/j.cels.2016.09.002) | [GSE85241](https://www.ncbi.nlm.nih.gov/geo/query/acc.cgi?acc=GSE85241) | [CEL-Seq2](http://dx.doi.org/10.1186/s13059-016-0938-8) | 9 | 2122 | 19140 |
| [Xin](http://dx.doi.org/10.1016/j.cmet.2016.08.018) | [GSE81608](https://www.ncbi.nlm.nih.gov/geo/query/acc.cgi?acc=GSE81608) | [SMARTer](http://www.clontech.com/US/Products/cDNA_Synthesis_and_Library_Construction/Next_Gen_Sequencing_Kits/Total_RNA-Seq/Universal_RNA_Seq_Random_Primed) | 4 | 1492 | 39851 |

**Table S3.** Pancreas datasets and summary information after removing cells with low quality or unknown cell labels.
