# Supplementary material for: Evaluation of single-cell classifiers for single-cell RNA sequencing data sets
Source: Brief Bioinform. 2019 Oct 23;21(5):1581–95. doi: 10.1093/bib/bbz096 (PMC7947964; doi:10.1093/bib/bbz096)
Supplement: Table_S4_bbz096 [file table_s4_bbz096.docx]

| **Baron**  **/8569** | acinar | activated_stellate | alpha | beta | delta | ductal | endothelial |
| --- | --- | --- | --- | --- | --- | --- | --- |
|  | 958 | 284 | 2326 | 2525 | 601 | 1077 | 252 |
|  | epsilon | quiescent_stellate | gamma | mast | schwann | t_cell | macrophage |
|  | 18 | 173 | 255 | 25 | 13 | 7 | 55 |

| **Muraro**  **/2122** | acinar | alpha | beta | delta | ductal | endothelial | epsilon | gamma | mesenchymal |
| --- | --- | --- | --- | --- | --- | --- | --- | --- | --- |
|  | 219 | 812 | 448 | 193 | 245 | 21 | 3 | 101 | 80 |

| **Xin**  **/1492** | alpha | beta | delta | gamma |
| --- | --- | --- | --- | --- |
|  | 886 | 472 | 49 | 85 |

**Table S4.** Detailed information of three pancreas datasets including total cell counts and cell counts per cell type. Cell types with red font mean that they are new cell types, for instance, the mesenchymal is a new cell type to Baron dataset.
