# Supplementary material for: Evaluation of single-cell classifiers for single-cell RNA sequencing data sets
Source: Brief Bioinform. 2019 Oct 23;21(5):1581–95. doi: 10.1093/bib/bbz096 (PMC7947964; doi:10.1093/bib/bbz096)
Supplement: Table_S5_bbz096 [file table_s5_bbz096.docx]

| **Tools** | **Reasons of excluding** |
| --- | --- |
| **celaref** | Requires clustering process prior to classification. |
| **cardelino** | Designs for assigning donor and clone identities to individual cells from scRNA-seq data. |
| **Clustermap** | Requires clustering process prior to classification. |
| **Distmap** | Requires 3D gene expression data. |
| **DropLasso** | Determines features’ weights only. |
| **FateID** | Designs for the quantification of the fate biases of multipotent progenitors and requires clustering before classification. |
| **MetaNeighbour** | Requires cell labels or cluster annotation before classification. |
| **MIMOSCA** | Designs for Perturb-seq data. |
| **Moana** | Only provides a classifier for PBMC datasets. |
| **Para_DPMM** | Installation failed. It needs older version of gcc-4.7 and additional dependencies. |

**Table S5**. Classifications tools not evaluated in this paper and reasons of excluding.
