# Supplementary material for: Evaluation of single-cell classifiers for single-cell RNA sequencing data sets
Source: Brief Bioinform. 2019 Oct 23;21(5):1581–95. doi: 10.1093/bib/bbz096 (PMC7947964; doi:10.1093/bib/bbz096)
Supplement: Table_S6_bbz096 [file table_s6_bbz096.docx]

| **Tools** | | **Parameter** | **Default** |
| --- | --- | --- | --- |
| **scmap** | **scmapc2c** | w | 3 |
|  |  | threshold | 0.5 |
|  | **scmapc2clus** | threshold | 0.7 |
| **scMCA** | | numbers_plot | 3 |
| **scPred** | | n (number of PC) | 10 |
|  |  | threshold | 0.9 |
| **SingleR** | | p.threshold | 0.05 |
|  |  | numCores | SingleR.numCores |
| **Seurat** | |  |  |
| **CaSTLe** | | nFeatures | 100 |
| **scID** | | contamination | 0.05 |
| **AltAnalyze** | |  |  |
| **CellFishing** | | n_min_features | 10% |
|  |  | k (number of neighbors) | 10 |

Table S6. Main parameters and default values of all tools evaluated in this paper. The parameter numCores in SingleR is the number of cores used to calculate and SingleR.numCores function returns the total core number. In this paper, one core is used in all tests.
